# Adaptive Empathy: Empathic Response Selection as a Dynamic, Feedback-Based Learning Process

**DOI:** 10.3389/fpsyt.2021.706474

**Published:** 2021-07-22

**Authors:** Elena Kozakevich Arbel, Simone G. Shamay-Tsoory, Uri Hertz

**Affiliations:** ^1^Department of Psychology, University of Haifa, Haifa, Israel; ^2^Integrated Brain and Behavior Research Center, Haifa, Israel; ^3^Department of Cognitive Sciences, University of Haifa, Haifa, Israel

**Keywords:** empathy, cognitive empathy, online simulation, social cognition, learning, reward, decision-making

## Abstract

Empathy allows us to respond to the emotional state of another person. Considering that an empathic interaction may last beyond the initial response, learning mechanisms may be involved in dynamic adaptation of the reaction to the changing emotional state of the other person. However, traditionally, empathy is assessed through sets of isolated reactions to another's distress. Here we address this gap by focusing on adaptive empathy, defined as the ability to learn and adjust one's empathic responses based on feedback. For this purpose, we designed a novel paradigm of associative learning in which participants chose one of two empathic strategies (reappraisal or distraction) to attenuate the distress of a target person, where one strategy had a higher probability of relieving distress. After each choice, participants received feedback about the success of their chosen strategy in relieving the target person's distress, which they could use to inform their future decisions. The results show that the participants made more accurate choices in the adaptive empathy condition than in a non-social control condition, pointing to an advantage for learning from social feedback. We found a correlation between adaptive empathy and a trait measure of cognitive empathy. These findings indicate that the ability to learn about the effectiveness of empathic responses may benefit from incorporating mentalizing abilities. Our findings provide a lab-based model for studying adaptive empathy and point to the potential contribution of learning theory to enhancing our understanding of the dynamic nature of empathy.

## Introduction

Empathy allows us to share emotions and understand the mental and affective states of others. While definitions of empathy may vary, one of the main objectives of empathic capabilities is to be able to respond to the emotional state of another person in order to alleviate that person's distress (1). Empathy has been shown to play a major role in promoting well-being (2), enhancing parenting skills (3), and supporting emotional development (4). There is strong evidence that empathy is a fundamental contributor to other-oriented prosocial behavior (5). Indeed, Zaki and Williams (1) suggested that empathy is apparent in the interpersonal emotion regulation cycle, as the distressed target evokes an empathic reaction in the observer, who may thus help the suffering person. Although empathic reactions can be covert and not communicated to others, e.g., change in mood, emotions, and thoughts, they are often overt, e.g., detectable facial or body expression, verbal response, and are conveyed back to the target. While empathic reactions do not necessarily lead to action, in many contexts of empathic interactions between an empathizer and a distressed target, they are the driver of prosocial responses. Also, the empathic interaction does not necessarily end with the initial empathic response. After feedback from the target, an individual's empathic responses may change, generating a process we refer to as *adaptive empathy* (6). Since we focus on empathic responses which are manifested in social interactions over time, the covert empathic reactions are beyond our scope, and from now and on we will focus on overt responses only, i.e., responses that are communicated to the distressed target. We currently do not know how the adaptive empathy process unfolds and how it is related to other learning processes and to trait empathy. Here we set out to examine adaptive empathy as a unique facet of empathy.

Despite a long tradition of studying empathy in social interactions in the field of social psychology (7, 8), most known paradigms measuring empathy involve one-shot, passive observation of a suffering target. Current studies rely either on directly asking individuals to evaluate their trait empathy or to assess their state empathy (9). These studies facilitated the essential behavioral and neural differentiation of empathy components and provided several classifications of empathic abilities, the most prevalent of which is the distinction between emotional and cognitive empathy (10, 11). Emotional empathy includes sharing of another's emotions, as well as emotional contagion, a condition in which one feels emotions detected in others (12–15). Cognitive empathy involves mentalizing and identifying another's thoughts and feelings (16), understanding another's perspective (11), as well as inferring and attributing mental states or traits to specific persons (17, 18). Mentalizing, also known as Theory of Mind (ToM), is important because of the assumption that other people's mental states determine their actions and influence their interactions (18, 19). Mentalizing is affected by culture and developmental stage (17, 20) and requires high-order cognitive abilities, such as cognitive flexibility (21, 22) and episodic memory (23). Both empathy components (emotional and cognitive) appear to operate independently on behavioral and neural levels, while an empathic response may encompass both processes or either one, depending on the context (11, 24). Notably, both types of empathy may affect the dynamic process of adaptive empathy. The sharing of another's emotional state serves as a trigger for the empathic interaction, hence, emotional empathy may be essential in contexts that include affective empathic responses such as empathic touch and facial expressions (25). By means of mentalizing the state of the distressed person, cognitive empathy may help the empathizer evaluate the effectiveness of responses before reacting and thus choose the appropriate response for the specific person in distress, or learn the most effective one over time. Cognitive empathy may therefore be most relevant in contexts where one suggests emotional regulation strategies to alleviate distress, using verbal communication for example, which is the context of the current experiments.

In line with the view that empathic responses are dynamic and adapted to the needs of the target, Shamay-Tsoory and Hertz (6) proposed examining empathy in the context of interactions between empathizer and target over time. Adaptive empathy is the process through which an empathizer detects the effects of his or her initial empathic response and adapts this response accordingly, i.e., learns what is the most effective response strategy. The core of this approach sees empathy as taking place along a feedback cycle, in which the probability of providing a specific empathic response changes within an interaction according to the feedback (9, 16, 26). This cycle can endure over multiple incidents of distress relief during an interpersonal (27) or therapeutic relationship (28). This feedback cycle is akin to many other well-studied learning paradigms (29, 30). Considering that the empathic response aims to diminish distress (2, 3), learning mechanisms may be involved in dynamic adaptation and tailoring of the response to the specific person we interact with. Learning in the social domain bears some similarities to learning in a non-social domain in terms of the general computations that drive learning, though social learning has also been shown to operate differently (16, 31). For example, when playing against humans as opposed to computers, participants preferred generosity over maximizing their reward (32). Moreover, recent evidence suggests that decisions in a social context are made by integrating multiple types of inferences about one's own rewards, others' rewards, and others' mental states (33, 34). Social learning processes have also been shown to be related to trait empathy. For example, high cognitive empathy correlated with the dynamics of learning about options that maximize rewards for others (35) and with increased prosocial tendencies (36). Moreover, higher levels of cognitive trait empathy predicted better emotion regulation by a long-term romantic partner, suggesting that the ability to understand the partner's point of view, i.e., mentalization, is an important factor in distress relief (37).

Here we aim to characterize adaptive empathy as a learning process. Our first goal was to compare adaptive empathy to other types of learning in terms of accuracy. Our main hypothesis was that during adaptive empathy participants will demonstrate an overall learning pattern resembling other statistical learning paradigms. Nevertheless, we also had a non-directional hypothesis, according to which learning the empathic responses would be distinct from non-social learning. We further sought to evaluate the relationship between adaptive empathy and traditional cognitive and emotional empathy measures. Since adjusting the empathy reaction in response to feedback must involve cognitive empathy elements, such as mentalizing and inference of the other's mental state, we hypothesized that in the adaptive empathy condition, but not in other conditions, learning accuracy would be associated with cognitive empathy. We further assumed that performance in the adaptive empathy condition would not be correlated with emotional empathy.

To test these hypotheses, we developed a novel experimental paradigm of two-choice associative learning, as an adaptation of the classical behavioral paradigm “two-armed bandit task.” In this task the participants must make repeated choices among options (bandit arms), learning about the statistical relations between choices and expected outcomes. Such tasks are often used in learning and decision-making studies, demonstrating the abilities of participants to learn about the most rewarding action and adjust their behavior accordingly (29, 38–41). In our paradigm, over multiple encounters, on each trial participants chose one of two empathic strategies (reappraisal or distraction) to attenuate the distress of a target. Following each choice, they observed the effect of their empathic response on the target's emotional state, such that the feedback could inform their future decisions. To pinpoint differences between empathic learning and other types of learning, participants also completed two control conditions involving learning about targets' food preference (social control) and the likely location of a monetary reward (non-social control). This paradigm allowed us to evaluate the relationship between adaptive empathy and learning in other contexts, and control for non-social associative learning skills, as well as assess the link between adaptive empathy and the individual's trait empathy.

## Materials and Methods

### Participants

For the study, which was conducted online, we recruited 199 participants [77 male, aged 39.3 ± 14 (mean ± std); 121 female, aged 35.2 ± 13.4] using the Prolific platform (December 16, 2020). The study was approved by the University of Haifa, Faculty of Social Sciences Research Ethics Committee (Project ID Number: 100/21), and the experiment was conducted in accordance with relevant guidelines and regulations. All participants were screened for neurological disorders. Due to technical issues, choice data were corrupted for 15 participants and therefore discarded in further data analysis. Furthermore, 21 participants were excluded from the study due to insufficient effort invested in the task: failure to complete the task within a reasonable time limit (inactive over half an hour during the task); always selected the same side or the same option; performance below 30% accuracy in one of the three blocks. This level of performance was chosen to avoid excluding participants that had difficulties in learning in one of the blocks, which are meaningful and relevant to our expected differences. Therefore, our final sample size for the analysis was *n* = 163. This sample size was sufficient to allow detection of a moderate effect size of individual difference (ρ = 0.2, β = 0.8).

### Adaptive Empathy Task

**Figure 1:**
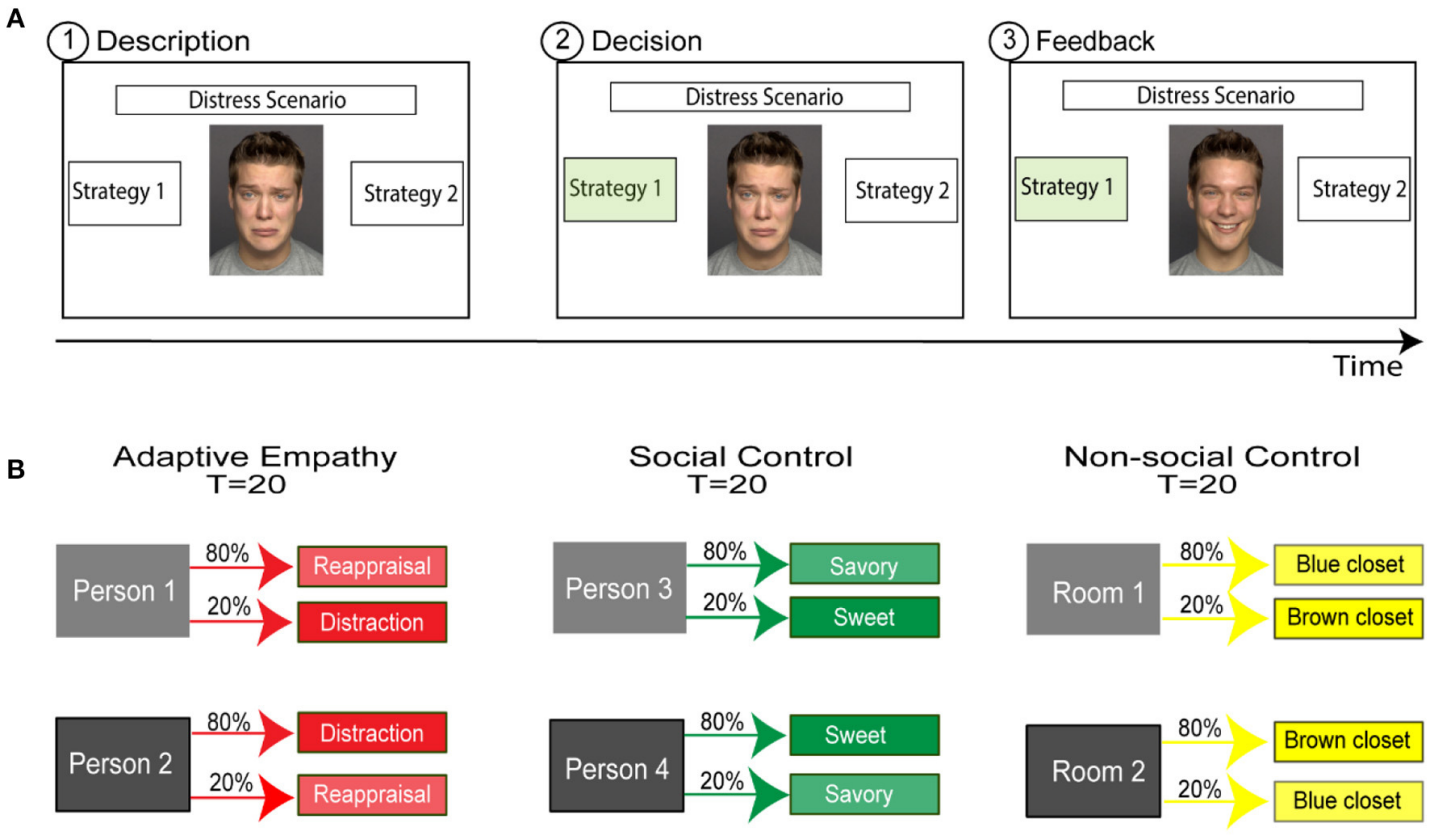
Experimental Design. **(A)** A sample trial in the adaptive empathy condition. Participants had to choose between two options and learn which one was more likely to lead to a desirable outcome. Each trial consisted of three stages: (1) Participants were shown a picture of a person with a sad facial expression, together with a textual description of the current cause of the person's distress. Textual descriptions of two empathic responses corresponding to two different emotion regulation strategies were also provided. (2) Participants chose one of two responses. (3) Feedback was provided regarding the effect of the chosen strategy, as indicated by the person's face changing to a happy expression or remaining sad. **(B)** Overall experimental design of the adaptive empathy task. The task included three experimental learning conditions carried out by all participants. In each condition, participants learned about one person/room. The order of the blocks and the preferred strategy learned in each block were randomized across participants.

In the adaptive empathy task, the paradigm included three conditions: adaptive empathy, social control, and non-social control. Each condition included 20 trials in which participants had to choose between two options and learn which is more likely to lead to a desirable outcome (Figure 1). In each condition, the participant interacted with one person/room over 20 trials. For example, a participant could make 20 decisions to alleviate person 1 distress in the adaptive empathy condition, 20 decisions regarding food courses for person 3 in the social-control condition, and 20 closet choices in room 2 in the non-social condition. The targets in each condition were counterbalanced across participants. The gender of the target person matched the participant's gender. The order of the conditions was randomized between participants. Progress within and between the trials was self-paced. The task was developed using JS and HTML (see Figure 1A for sample screens, the code is freely available in the Open Science Framework https://osf.io/dgt5e/).

#### Adaptive Empathy Condition

In this condition, participants were shown 20 distress-related scenarios entailing a target person. Each trial began with a picture of the person with a sad facial expression, alongside a textual description of the current cause of the person's distress (description stage) (e.g., “Ben and his girlfriend broke up”). While viewing the scenario, participants were instructed to select one of two responses aimed at diminishing the distress of the targets. The responses were two types of emotion regulation strategies (distraction vs. reappraisal): “Let's go camping on the beach, maybe set up a campfire and take a swim” (distraction strategy) or “The relationship depends on both of you; maybe she just needed some me time” (reappraisal strategy). Finally, the participant observed the effect of the chosen strategy, indicated by the person's face changing to a happy expression or remaining sad (feedback stage). Unbeknownst to the participants, one strategy was more likely to relieve the target, with a probability of 0.8, while the other strategy had a relief probability of 0.2. About half the participants (86) interacted with a target that preferred reappraisal, while 77 participants interacted with a target that preferred distraction (see Figure 1B).

#### Social Control Condition

In this control condition, the participant was required to learn about a target person's food preferences over 20 trials. Each trial began with a picture of the person with a neutral facial expression, alongside a textual description of a restaurant where the participants were supposedly present. The participant was offered two types of dishes (savory main course and sweet dessert), e.g., “Chop steak freshly ground and smothered with grilled mushrooms, onions and savory garlic sauce” or “Crepes with Nutella, strawberry, cherry, apple or apricot rich jam and ice cream,” and had to choose one that would please the target. Finally, the participant observed the effect of the chosen dish on the target, as indicated by the target's face changing to a happy expression or remaining neutral (feedback stage). One type of dish had a higher probability (*p* = 0.8) of pleasing the target, while the other had a low probability of pleasing the target (*p* = 0.2; see Figure 1B).

#### Non-social Control Condition

In this control condition, the participant was required to learn which of two closets is more likely to contain a monetary reward over 20 trials. On each trial, after selecting a closet, the participant observed the effect of the choice (closet), indicated by whether the opened closet contained the money or was empty (feedback stage). One closet was more likely to contain the monetary reward than the other (*p* = 0.8 vs. *p* = 0.2) (see Figure 1B).

### Paradigm and Stimuli

The facial stimuli shown to each participant were taken from the FACES Life Span Database of Facial Expressions, with their obtained permission (42). Only neutral, sad, and happy facial expressions for younger men and women were selected from the database.

The distress scenarios were taken from everyday life situations related to relationships, work, daily routines, and the like. The choice of emotion regulation strategies was based on a wide range of studies suggesting that cognitive reappraisal and expressive suppression (distraction) are widely used as emotion regulation strategies. *Reappraisal* is defined as changing the way one thinks about a situation, thus changing its emotional impact, while *distraction* is a strategy that involves inhibiting the emotion (43–46). The restaurant types were chosen according to popular categories found online.^1^ The dish descriptions were taken and adjusted from various online restaurant menus, according to the type of restaurant.

To create a similar reading load, all the strategies (emotion regulation and dish descriptions) consisted of 15 words on average. The stimuli were tested and confirmed in a pilot study with independent reviewers.

### Questionnaire of Cognitive and Affective Empathy

Based on a contemporary theoretical model of empathy, we chose the Questionnaire of Cognitive and Affective Empathy [QCAE; (47)] as the tool to assess participants' levels of trait cognitive and affective (emotional) empathy. The QCAE consists of 31 items grouped into two scales of cognitive and affective (emotional) empathy. The cognitive empathy (CE) scale includes two subscales: *perspective taking* (PT) - the ability to see a situation from another person's perspective (e.g., “I can easily tell if someone else wants to enter a conversation”); *online simulation* (OS) - the ability to understand and mentally represent or imagine how another person is feeling (e.g., “Before criticizing somebody, I try to imagine how I would feel if I was in their place”). The affective empathy (AE) scale includes three subscales: *emotion contagion* (EC) - the automatic mirroring of emotions of others (e.g., “I am happy when I am with a cheerful group and sad when the others are glum”); *peripheral responsivity* (PER)—the emotional reaction to the mental states of others in a detached social context (e.g., “I often get deeply involved with the feelings of a character in a film, play, or novel”); and *proximal responsivity* (4 items)—the emotional reaction to the moods of others in a physically or emotionally close social context (e.g., “I often get emotionally involved with my friends' problems”). Items are rated on a 4-point Likert scale ranging from 1 = “strongly disagree” to 4 = “strongly agree.” Higher scores indicate greater empathy.

### Procedure

Participants were recruited using the Prolific platform and performed the experimental task online on their own computers, using a mouse to input their choices (smartphones or similar devices were blocked). They began by reading information about the experiment, signing an informed consent form, and answering several demographic questions (age, gender, and level of education). The participants were paid a fixed monetary compensation of £4 for their participation and were promised a performance-based bonus of £1 maximum for making correct choices across all experimental conditions. The central part of the experiment, i.e., the Adaptive Empathy Task, followed. The task average duration across participants was 8.2 min (SD = 3.2 min; MIN = 4.2 min; MAX = 24.8 min). The durations per block are detailed in Supplementary Table 2.3. Upon completing the task, participants were asked to complete the empathy scales questionnaire (QCAE).

### Analysis

Statistical analyses were conducted using R version 4.0.1 (48), with the following packages: rstatix (49), afex (50), and jtools (51). Differences in accuracy between conditions were examined by a one-way repeated-measures ANOVA, followed-up by a *post-hoc* paired-samples *t*-test to determine the origin of the differences. A Welch *t*-test for unequal variances was conducted to compare means between two preferred strategies by different targets within each condition, considering two independent samples of participants receiving one of the two targets. To directly examine the relationship between adaptive empathy and trait empathy scales, we applied separate linear regression models. Participants' accuracy in each block, as well as the difference in accuracy between adaptive empathy and a non-social control block, served as dependent variables, while empathy scores served as independent variables.

## Results

### Learning Accuracy Between Conditions and According to Preferred Strategy

**Figure 2:**
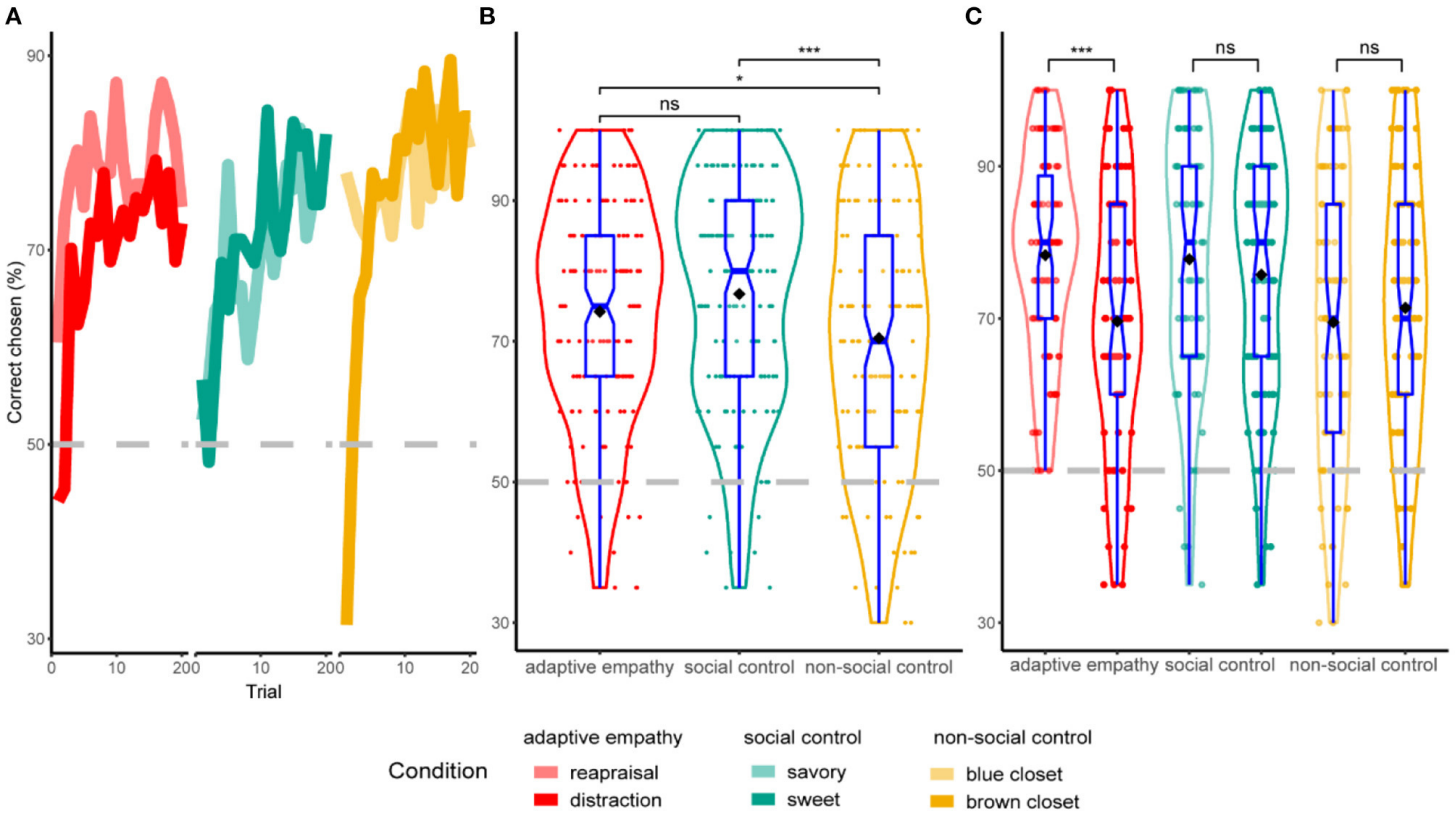
Learning accuracy between conditions. **(A)** Group-level learning curves showing choice behavior in the three learning conditions. Trials are averaged over the three conditions for adaptive empathy, social control, and non-social control. Dashed lines indicate chance level. **(B)** Comparison of accuracy between the three conditions. Participants exhibited significantly higher learning accuracy in social vs. non-social and in adaptive empathy vs. non-social control conditions. **(C)** Comparison of learning accuracy within each condition, between strategies preferred by the target. Participants exhibited significantly higher learning accuracy when learning that targets preferred reappraisal over distraction. *p ≤ 0.05, ***p ≤ 0.001, ns, not significant.

The participants performed on average above chance-level (50%), suggesting learning of emotion regulation preferences, food preferences, and money location (see Figure 2A). We also compared the learning accuracy between the conditions, applying a one-way repeated-measures ANOVA at three levels of a within-subjects variable block type (adaptive empathy, social control, and non-social control). This analysis revealed a significant difference in average learning accuracy between conditions [*F*_(2, 324)_ = 6.43^**^, *p* = 0.002, $\eta_{P}^{2}$ = 0.038]. Follow-up *post-hoc* paired *t*-tests showed that the highest accuracy emerged in the social control condition (M = 76.72, SD = 16.22), which was significantly higher than the accuracy levels in the non-social control condition [*t*_(162)_ = 3.73, *p* < 0.001, *d* = 0.29], which exhibited the lowest learning accuracy (M = 70.46, SD = 18.19). In line with our prediction, accuracy in the adaptive empathy condition (M = 74.20, SD = 15.64) was significantly higher than in the non-social condition [*t*_(162)_ = 2.03, *p* = 0.04, *d* = 0.16] (see Figure 2B). We further compared performance within the adaptive empathy condition, showing that the mean accuracy for the reappraisal strategy was 78.31 (SD = 13.25), whereas the mean accuracy for the distraction strategy was 69.61 (SD = 16.87). The Welch two-sample *t*-test showed that the difference was statistically significant, *t*_(143.9)_ = 3.633, *p* < 0.001, *d* = 0.57. No such differences were found between strategies in the other conditions (see Figure 2C).

### Relationship Between Trait Empathy and Adaptive Empathy

**Figure 3:**
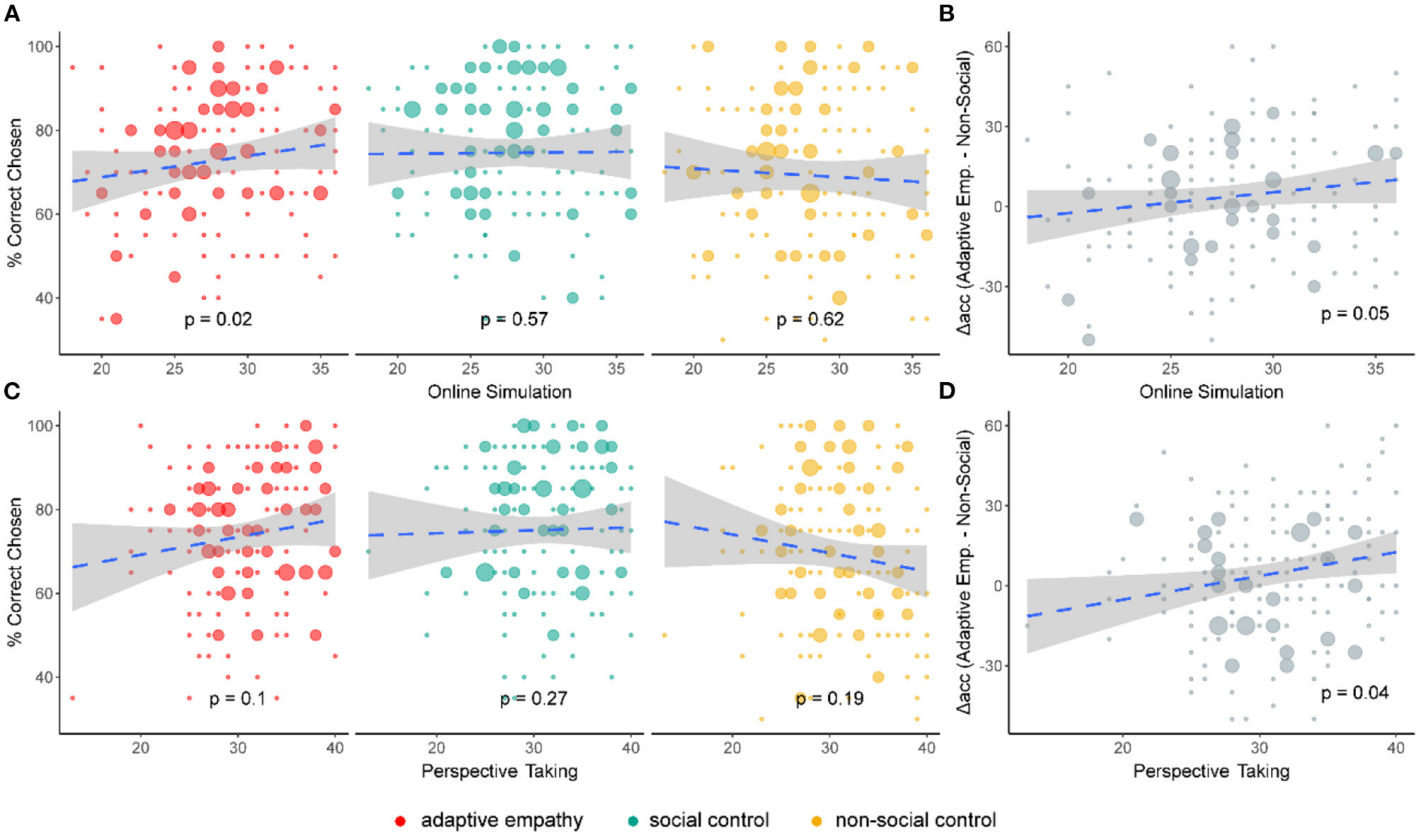
Cognitive empathy subscales. **(A)** Correlations between task conditions and cognitive empathy trait: participants high on the online simulation subscale exhibited higher performance in adaptive empathy. There was no such correlation in other conditions. **(B)** Correlation of difference in accuracy between adaptive empathy and non-social control with cognitive empathy trait: participants high in online simulation had a larger gap in accuracy between the two conditions. **(C)** Same as **(A)**, but for the perspective taking subscale. No correlation was found with performance in any of the conditions. **(D)** Same as **(B)**, but for the perspective taking subscale: participants high in perspective taking had a larger gap in accuracy between the two conditions. Dashed lines indicate the fitted linear regression, gray areas indicate a 95% confidence interval.

We tested whether individuals' cognitive empathy rates were uniquely associated with adaptive empathy. In separate linear regression analyses, the two cognitive empathy subscales were entered as potential predictor variables, gender, and age as control variables, and learning accuracy at each condition was entered as the single dependent variable (see Figure 3). Consistent with our predictions, the analyses revealed that the online simulation subscale (47), a measure of trait empathy that probes the tendency to understand and imagine how another person is feeling, was positively associated with learning accuracy in the adaptive empathy condition [β = 0.67 ± 0.28, *t*_(158)_ = 2.39, *p* = 0.02]. Such an association was not found for the social control and non-social control conditions, indicating that online simulation makes a unique contribution to adaptive empathy (see Figure 3A). We directly compared the difference in slopes between the adaptive empathy and non-social conditions, by subtracting each participant's accuracy in the adaptive empathy condition from the accuracy in the non-social condition, and regressing this difference against the cognitive subscales. The linear regression results showed that the difference in accuracy was significantly correlated with the online simulation subscale, such that those high in this subscale exhibited a larger gap in performance between adaptive empathy and non-social control conditions [β = 0.83 ± 0.43, *t*_(158)_ = 1.96, *p* = 0.05]. The perspective taking subscale was also positively correlated with the difference in accuracies between adaptive empathy and the non-social conditions [β = 0.75 ± 0.35, *t*_(158)_ = 2.12, *p* = 0.04] (see Figures 3B,D; Supplementary Results-Simple Linear Regression Tables in Supplementary Material).

**Figure 4:**
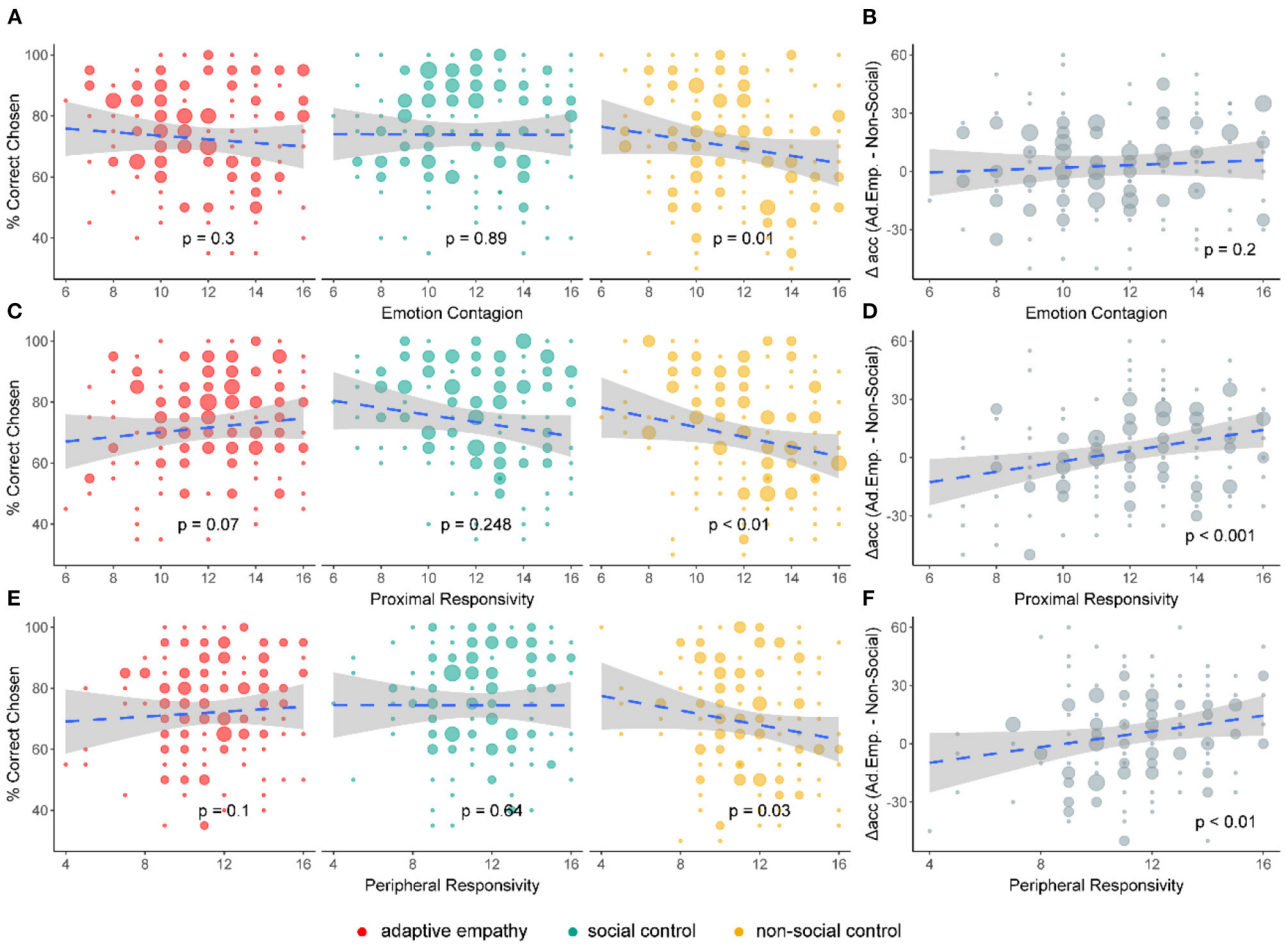
Emotional empathy subscales. **(A)** Correlations between task conditions and emotional empathy trait: participants high in emotional contagion exhibited a deficit in learning in the non-social control condition. No such deficit was found for other conditions. **(B)** Correlation of difference in accuracy between adaptive empathy and non-social control with emotional empathy trait: there was no gap between conditions across emotion contagion scores. **(C)** Same as **(A)**, but for the proximal responsivity subscale, showing a learning deficit in non-social control conditions for higher scores. **(D)** Same as **(B)**, but for the proximal responsivity subscale: participants high in proximal responsivity had a larger gap in accuracy between the two conditions. **(E)** Same as **(A)**, but for the peripheral responsivity subscale, showing a learning deficit in non-social control conditions for higher scores. **(F)** Same as **(B)**, but for the peripheral responsivity subscale: participants high in peripheral responsivity exhibited a larger gap in accuracy between the two conditions.

We conducted another set of linear regression analyses by entering the three emotional empathy subscales as potential predictor variables, gender and age as control variables, and learning accuracy in each condition as the single dependent variable (see Figure 4). No correlation was found between emotional empathy and performance in the adaptive empathy and social control conditions. However, emotional empathy scores - emotion contagion (EC), proximal responsivity (PRR), and peripheral responsivity (PER) - exhibited a negative association with learning accuracy in the non-social condition [β = −1.5 ± 0.57, *t*_(158)_ = −2.62, *p* = 0.01; β = −1.83 ± 0.59, *t*_(158)_ = −3.09, *p* = 0.002; β = −1.33 ± 0.6, *t*_(158)_ = −2.2, *p* = 0.03, respectively] (see Figures 4A,C,E; Supplementary Results-Simple Linear Regression Tables in Supplementary Material). In other words, higher levels of emotional empathy had a detrimental effect on learning in the non-social condition. Here, the PRR and PER subscale scores also predicted the difference between adaptive empathy accuracy and the non-social condition, such that higher trait empathy predicted a larger gap in accuracy between the conditions (see Figures 4D,F; Supplementary Results-Simple Linear Regression Tables in Supplementary Material).

## Discussion

The present study investigated adaptive empathy, i.e., the way participants learned and adapted their empathic responses according to the impact of these responses on a target person and the way this learning process corresponded with trait empathy measurements. We found a significant difference in choice accuracy between social and non-social conditions, as participants were more accurate in their choices of empathic responses and food preferences than in their choices of reward locations. This suggests that learning in the social domain is comparable to or even superior to non-social learning, even though the social domain involved more complex scenarios and option descriptions. Furthermore, within the adaptive empathy condition, performance was significantly higher when the target person preferred reappraisal rather than distraction. No such differences emerged in the other conditions. We observed an association between adaptive empathy and traditional empathy measures. In line with our hypothesis, the analysis revealed that cognitive empathy, and specifically its online simulation subscale, correlated with performance in the adaptive empathy condition only. The emotional empathy trait's subscales were not correlated with performance in the adaptive empathy condition, but were found to be negatively associated with performance in the non-social control condition. These results indicate that adaptive empathy is comparable to other learning processes and is linked to cognitive empathy abilities, at least when learning about the effectiveness of emotion regulation strategies. These findings suggest that adaptive empathy may be an important facet of empathy, which may influence the dynamics and outcomes of social interactions.

Our findings of higher accuracy levels in the social conditions support the idea that learning in the social domain is somewhat different than in other, non-social domains (34). The notion of privileged learning in the social domain has been examined from different perspectives, among them cognitive (i.e., which cognitive processes are involved in this process) and motivational (i.e., what are the goals or intentions of the learner) (52). From the cognitive perspective, while social and non-social learning may depend on the same basic learning mechanisms (53), learning about people may incorporate prior, human-specific, expectations, such as consistency of people's traits over time and expectations about how people may respond to different actions based on previous encounters and our own experiences (16, 54), which we do not use when learning about the location of money rewards (as an example). For instance, in the case of learning about people's moral behaviors, the attribution of selfish behavior to a person's character was found to be more volatile than the attribution of moral or prosocial behaviors (55). Such a bias was not observed when learning about the resource-sharing decisions of non-human agents. Hence, the distinction found in social vs. non-social learning may not be due to differences in basic learning mechanisms *per se*, but rather result from our mentalizing capacity or theory of mind, in the form of a socially specific cognitive module that is present when learning from a social partner (34, 56). Mentalizing and employing an internal model of human mind may make learning about other people, i.e., reasoning and forming predictions about them, easier than learning about abstract associations (16, 54).

The correlation observed here between cognitive empathy and performance in the adaptive empathy condition supports the role of mentalizing in social learning. Higher levels of cognitive empathy ability, and specifically its online simulation subscale, were linked with enhanced ability to adapt one's empathic response based on feedback from the target person. The online simulation subscale developed by Reniers et al. (47) is defined as the capacity to simulate other people's feelings and is relatively similar to perspective taking from the Interpersonal Reactivity Index [IRI; (57)]. However, as suggested by Heym et al. (58), this scale seems to encompass not only imagining how other people feel, but also how they think and may act, i.e., simulating other people's mental states (both thoughts and feelings) and spontaneously adopting their psychological point of view, which resembles the traditional conceptualization of mentalizing (17). Mentalizing may greatly aid the iterative process of interpersonal emotion regulation, i.e., adaptive empathy, as it involves learning and adjusting one's expectations of another person's behavior and determining which course of action will have a more relieving effect on a specific person (16–19). Moreover, a previous study showed that individuals with high scores on the online simulation subscale learned equally fast for the benefit of others as for their own benefit, as opposed to those who scored low on this subscale and learned slower for others (59). This is in line with our finding that people who scored high on the online simulation subscale are better in learning about emotion regulation preferences of others than are individuals with low scores on this subscale.

In addition to mentalizing, learning about humans integrates prior biases and assumptions (33). Such prior expectations about other people may explain the difference found in adaptive empathy accuracy between the two emotion regulation strategies, as reappraisal strategy was more readily learned than distraction strategy. The use of reappraisal rather than distraction to regulate emotions is widely considered to be associated with well-being. Researchers have also suggested that reappraisal is more effective and has healthier emotional, cognitive and social consequences than distraction (60, 61). Hence, learning that reappraisal rather than distraction is the most effective strategy may be easier due to common knowledge about the success of this strategy in coping with negative emotions (62). In addition, the scenarios presented to the participants were low-intensity distress situations. Previous findings showed that individuals prefer to regulate emotions using reappraisal in such situations, compared with high-intensity distress situations, in which they prefer to use distraction (43).

Another factor shaping social learning is motivation, which may also explain the differences in performance observed here. Although empathy is an effortful process that people sometimes tend to avoid (63, 64), it may still be affected by stronger motivational factors, e.g., approach motives (65), than the demand to find a monetary reward. Perhaps the evaluative feedback, e.g., the emotional response in the form of a smiling or sad face, is considered more valuable than a reward in the form of money in a closet (34). Our results indicate that those high in emotional empathy displayed lower performance in the abstract value-based condition, but when their learning was associated with people, their performance level remained intact. If we consider a target person's emotional responses as motivating learning, high emotional empathy may be more affected by the target's emotional responses. That is, the participant may be more affected by sad/happy facial expressions and more driven to learn the most effective strategy. Another motivation to learn in a social context may be the desire to maintain a social connection (66). According to the “Need-to-belong” theory (67), the motivation to form social relationships shapes cognition and behavior and may be an essential factor when operating in a social interaction context rather than in an abstract one. Studies show that people are willing to pay more to reduce the pain of others than to reduce their own pain (68).

Another possible explanation for differences in accuracy between social and non-social conditions, and for the negative correlation between non-social performance and emotional empathy levels, may be rooted in empathizing-systemizing theory (69). According to this theory, strong empathizers are good at understanding the social world. These individuals show an advantage in emotion recognition and social sensitivity, while strong systemizers are detail-oriented, good at understanding how things work and excel at solving technical problems. Previous research on social information processing by empathizers and systemizers revealed that empathizers, in contrast to systemizers, had stronger activation in brain areas related to emotional empathy during emotional empathy tasks (70). Our findings offer additional support for the fact that highly empathic individuals exhibit poor performance when it comes to problems in the physical world.

### Potential Limitations

The current study was designed to examine adaptive empathy empirically by means of a novel experimental task that allows comparison of the empathic learning process to other, well-established learning paradigms. As such, it uses a computerized task that is somewhat distanced from real-life social interactions. In such context, emotional empathy traits effect on adaptive empathy may be limited. It may therefore be that when using a face-to-face paradigm, where social cues and empathic responses such as touch, tone of voice, and facial expressions are available, emotional empathy may have a greater influence on adaptive empathy. Another limitation has to do with the non-social condition used here. This condition was designed to be similar to learning paradigms in the non-social literature. It differed both in its abstract action-outcome association of money in closets compared with the more concrete social conditions (food leads to satisfaction, emotion regulation leads to distress relief) and in the cognitive demands of recognizing the different strategies. The adaptive empathy condition involves a demanding request to detect different empathic response strategies presented in text, and the food choices were menu items presented in text, and the underlying strategy (distraction/reappraisal or savory/sweet) had to be inferred. However, in line with previous studies, in the non-social condition, the participant had to choose between two closets, which were identical in each trial. The finding that accuracy was lower in the non-social condition may therefore stem from participants finding the social conditions more engaging. While we address the effect of motivation in the social conditions, highlighting the negative correlation of emotional empathy and accuracy in the non-social condition, and we use additional social-control condition, future studies should aspire to use more engaging non-social control conditions. Future studies may adapt our current task to track specific aspects of adaptive empathy, such as differentiating the roles of expectations and motivation in adaptive empathy and studying it in different contexts.

## Conclusion

This research provides a new approach to viewing empathy as a dynamic, feedback-based process. Taking the dynamic dimension of empathy into account can enhance our understanding of the empathy construct, for example by examining the relationship between adaptive empathy and other prosocial and empathic skills, such as prosocial learning and prosocial tendencies. Our work indicates that adaptive empathy is indeed comparable to other learning processes, and therefore future studies may draw on the vast body of findings, paradigms, and models used in learning research to better characterize this process. In addition, adaptive empathy was linked with trait empathy measures. Such a link may be useful in examining how the social deficits present in different psychopathologies are related to aspects of the adaptive process, for example, due to malfunctioning in emotional identification or mentalization.

## Data Availability Statement

The datasets presented in this study can be found in online repositories. The names of the repository/repositories and accession number(s) can be found below: the Open Science Framework (https://osf.io/dgt5e/).

## Ethics Statement

The studies involving human participants were reviewed and approved by The University of Haifa, Faculty of Social Sciences Research Ethics Committee. The participants provided their written informed consent to participate in this study.

## Author Contributions

EK, SS-T, and UH: conceptualization, writing–review, and editing. EK: analysis, methodology, software, and writing–original draft. SS-T and UH: supervision. EK and UH: visualization. All authors contributed to the article and approved the submitted version.

## Conflict of Interest

The authors declare that the research was conducted in the absence of any commercial or financial relationships that could be construed as a potential conflict of interest.

## Footnotes

^1^https://en.wikipedia.org/wiki/Category:Restaurants_by_type

## References

[1] Zaki J Williams WC. Interpersonal emotion regulation. Emotion. (2013) 13:803–10. 10.1037/a0033839 24098929

[2] Morelli SA Lieberman MD Zaki J. The emerging study of positive empathy. Soc Personal Psychol Compass. (2015) 9:57–68. 10.1111/spc3.12157

[3] Atzil S Gao W Fradkin I Barrett LF. Growing a social brain. Nat Hum Behav. (2018) 2:624–36. 10.1038/s41562-018-0384-6 31346259

[4] Gonzalez-Liencres C Shamay-Tsoory SG Brüne M. Towards a neuroscience of empathy: ontogeny, phylogeny, brain mechanisms, context and psychopathology. Neurosci Biobehav Rev. (2013) 37:1537–48. 10.1016/j.neubiorev.2013.05.001 23680700

[5] Eisenberg N Eggum ND Di Giunta L. Empathy-related responding: associations with prosocial behavior, aggression, and intergroup relations. Soc Issues Policy Rev. (2010) 4:143–80. 10.1111/j.1751-2409.2010.01020.x 21221410 PMC3017348

[6] Hertz U Shamay-Tsoory S. Adaptive empathy: a model for learning empathic responses based on feedback. PsyArXiv. (2021) [Preprint]. 10.31234/osf.io/juc87 35050819

[7] Thomas G Fletcher GJO Lange C. On-line empathic accuracy in marital interaction. J Pers Soc Psychol. (1997) 72:839–50. 10.1037/0022-3514.72.4.839

[8] Stinson L Ickes W. Empathic accuracy in the interactions of male friends versus male strangers. J Pers Soc Psychol. (1992) 62:787–97. 10.1037/0022-3514.62.5.787 1593418

[9] Main A Walle EA Kho C Halpern J. The interpersonal functions of empathy: a relational perspective. Emot Rev. (2017) 9:358–66. 10.1177/1754073916669440

[10] Eres R Decety J Louis WR Molenberghs P. Individual differences in local gray matter density are associated with differences in affective and cognitive empathy. Neuroimage. (2015) 117:305–310. 10.1016/j.neuroimage.2015.05.038 26008886

[11] Shamay-Tsoory SG. The neural bases for empathy. Neurosci. (2011) 17:18–24. 10.1177/1073858410379268 21071616

[12] Zaki J Ochsner K. The neuroscience of empathy: progress, pitfalls and promise. Nat Neurosci. (2012) 15:675–80. 10.1038/nn.3085 22504346

[13] Hatfield E Cacioppo JT Rapson RL. Emotional Contagion Cambridge University Press (1993). 10.1017/CBO9781139174138

[14] Bernhardt BC Singer T. The neural basis of empathy. Annu Rev Neurosci. (2012) 35:1–23. 10.1146/annurev-neuro-062111-150536 22715878

[15] Jospe K Flöe A Lavidor M. The interaction between embodiment and empathy in facial expression recognition. Soc Cogn Affect Neurosci. (2018) 13:203–15. 10.1093/scan/nsy005 29378022 PMC5827354

[16] Tamir DI Thornton MA. Modeling the predictive social mind. Trends Cogn Sci. (2018) 22:201–12. 10.1016/j.tics.2017.12.005 29361382 PMC5828990

[17] Frith U Frith CD. Development and neurophysiology of mentalizing. Philos Trans R Soc London Ser B Biol Sci. (2003) 358:459–73. 10.1098/rstb.2002.1218 12689373 PMC1693139

[18] Premack D Woodruff G. Does the chimpanzee have a theory of mind? Behav Brain Sci. (1978) 1:515–26. 10.1017/S0140525X00076512

[19] Frith CD Frith U. The neural basis of mentalizing. Neuron. (2006) 50:531–4. 10.1016/j.neuron.2006.05.001 16701204

[20] Heyes CM Frith CD. The cultural evolution of mind reading. Science. (2014) 344:1243091. 10.1126/science.1243091 24948740

[21] Decety J Jackson PL. The functional architecture of human empathy. Behav Cogn Neurosci Rev. (2004) 3:71–100. 10.1177/1534582304267187 15537986

[22] Shamay-Tsoory SG Tomer R Goldsher D Berger BD Aharon-Peretz J. Impairment in cognitive and affective empathy in patients with brain lesions: anatomical and cognitive correlates. J Clin Exp Neuropsychol. (2004) 26:1113–27. 10.1080/13803390490515531 15590464

[23] Perry D Hendler T Shamay-Tsoory SG. Projecting memories: the role of the hippocampus in emotional mentalizing. Neuroimage. (2011) 54:1669–76. 10.1016/j.neuroimage.2010.08.057 20817106

[24] Shamay-Tsoory SG Aharon-Peretz J Perry D. Two systems for empathy: a double dissociation between emotional and cognitive empathy in inferior frontal gyrus versus ventromedial prefrontal lesions. Brain. (2009) 132:617–27. 10.1093/brain/awn279 18971202

[25] Englis BG Vaughan KB Lanzetta JT. Conditioning of counter-empathetic emotional responses. J Exp Soc Psychol. (1982) 18:375–91. 10.1016/0022-1031(82)90060-9

[26] Zaki J. Empathy: a motivated account. Psychol Bull. (2014) 140:1608–47. 10.1037/a0037679 25347133

[27] Goldstein P Weissman-Fogel I Dumas G Shamay-Tsoory SG. Brain-to-brain coupling during handholding is associated with pain reduction. Proc Natl Acad Sci. (2018) 115:E2528–37. 10.1073/pnas.1703643115 29483250 PMC5856497

[28] Barrett-Lennard GT. The empathy cycle: refinement of a nuclear concept. J Couns Psychol. (1981) 28:91–100. 10.1037/0022-0167.28.2.91

[29] Lockwood PL Klein-Flügge MC. Computational modelling of social cognition and behaviour-a reinforcement learning primer. Soc Cogn Affect Neurosci. (2020) nsaa040. 10.1093/scan/nsaa040 32232358 PMC8343561

[30] Zhang L Lengersdorff L Mikus N Gläscher J Lamm C. Using reinforcement learning models in social neuroscience: frameworks, pitfalls and suggestions of best practices. Soc Cogn Affect Neurosci. (2020) 15:695–707. 10.1093/scan/nsaa089 32608484 PMC7393303

[31] Farmer H Hertz U Hamilton AFC. The neural basis of shared preference learning. Soc Cogn Affect Neurosci. (2019) 14:1061–1072. 10.1093/scan/nsz076 31680152 PMC6970152

[32] Hackel LM Mende-Siedlecki P Amodio DM. Reinforcement learning in social interaction: the distinguishing role of trait inference. J Exp Soc Psychol. (2020) 88:103948. 10.1016/j.jesp.2019.103948

[33] Suzuki S Adachi R Dunne S Bossaerts P O'Doherty JP. Neural mechanisms underlying human consensus decision-making. Neuron. (2015) 86:591–602. 10.1016/j.neuron.2015.03.019 25864634 PMC4409560

[34] Ho MK MacGlashan J Littman ML Cushman F. Social is special: a normative framework for teaching with and learning from evaluative feedback. Cognition. (2017) 167:91–106. 10.1016/j.cognition.2017.03.006 28341268

[35] Lockwood PL. The anatomy of empathy: vicarious experience and disorders of social cognition. Behav Brain Res. (2016) 311:255–66. 10.1016/j.bbr.2016.05.048 27235714 PMC4942880

[36] Lockwood PL Seara-Cardoso A Viding E. Emotion regulation moderates the association between empathy and prosocial behavior. PLoS ONE. (2014) 9:e96555. 10.1371/journal.pone.0096555 24810604 PMC4014517

[37] Levy-Gigi E Shamay-Tsoory SG. Help me if you can: evaluating the effectiveness of interpersonal compared to intrapersonal emotion regulation in reducing distress. J Behav Ther Exp Psychiatry. (2017) 55:33–40. 10.1016/j.jbtep.2016.11.008 27888748

[38] Hertz U Bahrami B Keramati M. Stochastic satisficing account of confidence in uncertain value-based decisions. PLoS ONE. (2018) 13:1–23. 10.1371/journal.pone.0195399 29621325 PMC5886535

[39] Niv Y. Learning task-state representations. Nat Neurosci. (2019) 22:1544–53. 10.1038/s41593-019-0470-8 31551597 PMC7241310

[40] Behrens TEJ Woolrich MW Walton ME Rushworth MFS. Learning the value of information in an uncertain world. Nat Neurosci. (2007) 10:1214–21. 10.1038/nn1954 17676057

[41] Daw ND O'Doherty JP Dayan P Seymour B Dolan RJ. Cortical substrates for exploratory decisions in humans. Nature. (2006) 441:876–9. 10.1038/nature04766 16778890 PMC2635947

[42] Ebner NC Riediger M Lindenberger U. FACES-a database of facial expressions in young, middle-aged, and older women and men: development and validation. Behav Res Methods. (2010) 42:351–62. 10.3758/BRM.42.1.351 20160315

[43] Sheppes G Scheibe S Suri G Gross JJ. Emotion-regulation choice. Psychol Sci. (2011) 22:1391–6. 10.1177/0956797611418350 21960251

[44] Marroquín B. Interpersonal emotion regulation as a mechanism of social support in depression. Clin Psychol Rev. (2011) 31:1276–90. 10.1016/j.cpr.2011.09.005 21983267

[45] Gross JJ John OP. Individual differences in two emotion regulation processes: implications for affect, relationships, and well-being. J Pers Soc Psychol. (2003) 85:348–62. 10.1037/0022-3514.85.2.348 12916575

[46] Webb TL Miles E Sheeran P. Dealing with feeling: a meta-analysis of the effectiveness of strategies derived from the process model of emotion regulation. Psychol Bull. (2012) 138:775–808. 10.1037/a0027600 22582737

[47] Reniers RLEP Corcoran R Drake R Shryane NM Völlm BA. The QCAE: a questionnaire of cognitive and affective empathy. J Pers Assess. (2011) 93:84–95. 10.1080/00223891.2010.528484 21184334

[48] R Core Team. R: A Language and Environment for Statistical Computing. (2020). Available online at: https://www.r-project.org/ (accessed July 6, 2021).

[49] Kassambara A. rstatix: Pipe-Friendly Framework for Basic Statistical Tests (2020). Available online at: https://cran.r-project.org/package=jtools (accessed July 6, 2021).

[50] Singmann H Bolker B Westfall J Aust F Ben-Shachar MS. afex: Analysis of Factorial Experiments (2021). Available online at: https://cran.r-project.org/package=afex (accessed July 6, 2021).

[51] Long JA. jtools: Analysis and Presentation of Social Scientific Data. (2020). Available online at: https://cran.r-project.org/package=jtools (accessed July 6, 2021).

[52] Lockwood PL Apps MAJ Chang SWC. Is there a ‘Social' brain? implementations and algorithms. Trends Cogn Sci. (2020) 24:802–13. 10.1016/j.tics.2020.06.011 32736965 PMC7501252

[53] Heyes C. What's social about social learning? J Comp Psychol. (2012) 126:193–202. 10.1037/a0025180 21895355

[54] Saxe R Kanwisher N. People thinking about thinking people: the role of the temporo-parietal junction in “theory of mind. Neuroimage. (2003) 19:1835–42. 10.1016/S1053-8119(03)00230-1 12948738

[55] Siegel JZ Mathys C Rutledge RB Crockett MJ. Beliefs about bad people are volatile. Nat Hum Behav. (2018) 2:750–6. 10.1038/s41562-018-0425-1 31406285

[56] Koster-Hale J Saxe R. Theory of mind: a neural prediction problem. Neuron. (2013) 79:836–48. 10.1016/j.neuron.2013.08.020 24012000 PMC4041537

[57] Davis MH. Measuring individual differences in empathy: evidence for a multidimensional approach. J Pers Soc Psychol. (1983) 44:113–26. 10.1037/0022-3514.44.1.113

[58] Heym N Firth J Kibowski F Sumich A Egan V Bloxsom CAJ. Empathy at the heart of darkness: empathy deficits that bind the dark triad and those that mediate indirect relational aggression. Front Psychiatry. (2019) 10:95. 10.3389/fpsyt.2019.00095 30930800 PMC6423894

[59] Lockwood PL Apps MAJ Valton V Viding E Roiser JP. Neurocomputational mechanisms of prosocial learning and links to empathy. Proc Natl Acad Sci. (2016) 113:9763–8. 10.1073/pnas.1603198113 27528669 PMC5024617

[60] Gross JJ. Emotion regulation in adulthood: timing is everything. Curr Dir Psychol Sci. (2001) 10:214–9. 10.1111/1467-8721.00152

[61] John OP Gross JJ. Healthy and unhealthy emotion regulation: personality processes, individual differences, and life span development. J Pers. (2004) 72:1301–34. 10.1111/j.1467-6494.2004.00298.x 15509284

[62] Nezlek JB Kuppens P. Regulating positive and negative emotions in daily life. J Pers. (2008) 76:561–80. 10.1111/j.1467-6494.2008.00496.x 18399953

[63] Lockwood PL Hamonet M Zhang SH Ratnavel A Salmony FU Husain M. Prosocial apathy for helping others when effort is required. Nat Hum Behav. (2017) 1:1–10. 10.1038/s41562-017-0131 28819649 PMC5555390

[64] Contreras-Huerta LS Pisauro MA Apps MAJ. Effort shapes social cognition and behaviour: a neuro-cognitive framework. Neurosci Biobehav Rev. (2020) 118:426–39. 10.1016/j.neubiorev.2020.08.003 32818580

[65] Weisz E Zaki J. Motivated empathy: a social neuroscience perspective. Curr Opin Psychol. (2018) 24:67–71. 10.1016/j.copsyc.2018.05.005 29966924

[66] Pickett CL Gardner WL Knowles M. Getting a cue: the need to belong and enhanced sensitivity to social cues. Personal Soc Psychol Bull. (2004) 30:1095–107. 10.1177/0146167203262085 15359014

[67] Baumeister RF. Need-to-belong theory. In: Handbook of Theories of Social Psychology. London: SAGE Publications Ltd (2012). p. 121–40.

[68] Crockett MJ Kurth-Nelson Z Siegel JZ Dayan P Dolan RJ. Harm to others outweighs harm to self in moral decision making. Proc Natl Acad Sci. (2014) 111:17320–5. 10.1073/pnas.1408988111 25404350 PMC4260587

[69] Baron-Cohen S. Autism: the empathizing-systemizing (E-S) theory. Ann N Y Acad Sci. (2009) 1156:68–80. 10.1111/j.1749-6632.2009.04467.x 19338503

[70] Riekki T Svedholm-Häkkinen AM Lindeman M. Empathizers and systemizers process social information differently. Soc Neurosci. (2018) 13:616–27. 10.1080/17470919.2017.1368700 28826336

