# Supplementary material for: Adaptive Empathy: Empathic Response Selection as a Dynamic, Feedback-Based Learning Process
Source: Front Psychiatry. 2021 Jul 22;12:706474. doi: 10.3389/fpsyt.2021.706474 (PMC8339423; doi:10.3389/fpsyt.2021.706474)
Supplement: Supplementary file 1 [file Data_Sheet_1.docx]

Supplementary Material

Adaptive Empathy: Empathic response selection as a dynamic, feedback-based learning process

Elena Kozakevich Arbel^1*^, Simone G. Shamay-Tsoory^1, 2^, Uri Hertz^2, 3^

^1^Department of Psychology, University of Haifa, Haifa, Israel

^2^Integrated Brain and Behavior Research Center (IBBRC), Haifa

^3^Department of Cognitive Sciences, University of Haifa, Haifa, Israel

*** Correspondence:**Elena Kozakevich Arbel
elen.kozakevich@gmail.com

# Supplementary Methods

## Emotion Regulation Questionnaire

The Emotion Regulation Questionnaire (ERQ; 1) assesses individual differences in the habitual use of two common emotion regulation strategies: emotional suppression/distraction (4 items) and cognitive reappraisal (6 items). The ten items are rated on a 7-point Likert scale from 1= “strongly disagree” to 7= “strongly agree.” The reappraisal dimension contains items such as “I control my emotions by changing the way I think about the situation I’m in.” The suppression dimension has items such as “I control my emotions by not expressing them”.

# Supplementary Results

## Linear Regression Tables

Below are results of linear model fitting of learning accuracies in the three experimental blocks and the subscales of the Questionnaire of Cognitive and Affective Empathy (QCAE; 2): *perspective taking* (PT) - the ability to see the situation from another person’s perspective and *online simulation* (OS) - the ability to understand and mentally represent or imagine how another person is feeling (comprising Cognitive Empathy (CE) scale); *emotion contagion* (EC) - the automatic mirroring of emotions of others, *peripheral responsivity* (PER) – the emotional reaction to the mental states of others in a detached social context and *proximal responsivity* (4 items) – the emotional reaction to the moods of others in a physically or emotionally close social context (comprising the Affective Empathy (AE) scale).

Accuracy in adaptive empathy ~ Online Simulation score

F(4,158) = 2.37, p = 0.05, R² = 0.06, Adj. R² = 0.03

| Name | Estimate | 5% | 95% | t(158) | p |
| --- | --- | --- | --- | --- | --- |
| (Intercept) | 54.32 | 41.00 | 67.64 | 6.75 | 0.00 |
| OS | 0.67 | 0.21 | 1.13 | 2.39 | 0.02 |
| age | -0.01 | -0.16 | 0.14 | -0.06 | 0.95 |
| gender - male | 3.50 | -0.66 | 7.67 | 1.39 | 0.17 |
| gender - non-binary | 23.46 | -2.14 | 49.06 | 1.52 | 0.13 |

Accuracy in social control ~ Online Simulation score

F(4,158) = 2.46, p = 0.05, R² = 0.06, Adj. R² = 0.03

| Name | Estimate | 5% | 95% | t(158) | p |
| --- | --- | --- | --- | --- | --- |
| (Intercept) | 80.52 | 66.72 | 94.31 | 9.66 | 0.00 |
| OS | 0.16 | -0.32 | 0.64 | 0.57 | 0.57 |
| age | -0.26 | -0.42 | -0.11 | -2.79 | 0.01 |
| gender - male | 3.13 | -1.19 | 7.44 | 1.20 | 0.23 |
| gender - non-binary | 18.86 | -7.65 | 45.37 | 1.18 | 0.24 |

Accuracy in non-social control ~ Online Simulation score

F(4,158) = 1.16, p = 0.33, R² = 0.03, Adj. R² = 0.00

| Name | Estimate | 5% | 95% | t(158) | p |
| --- | --- | --- | --- | --- | --- |
| (Intercept) | 79.24 | 63.53 | 94.95 | 8.34 | 0.00 |
| OS | -0.16 | -0.71 | 0.38 | -0.50 | 0.62 |
| age | -0.16 | -0.33 | 0.02 | -1.48 | 0.14 |
| gender - male | 4.22 | -0.70 | 9.13 | 1.42 | 0.16 |
| gender - non-binary | 10.27 | -19.93 | 40.47 | 0.56 | 0.57 |

Accuracy in Δ(adaptive empathy – non-social control) ~ Online Simulation score

F(4,158) = 1.57, p = 0.19, R² = 0.04, Adj. R² = 0.01

| Name | Estimate | 5% | 95% | t(158) | p |
| --- | --- | --- | --- | --- | --- |
| (Intercept) | -24.92 | -45.19 | -4.65 | -2.03 | 0.04 |
| OS | 0.83 | 0.13 | 1.54 | 1.96 | 0.05 |
| age | 0.15 | -0.08 | 0.38 | 1.11 | 0.27 |
| gender - male | -0.72 | -7.06 | 5.62 | -0.19 | 0.85 |
| gender - non-binary | 13.20 | -25.76 | 52.15 | 0.56 | 0.58 |

Accuracy in adaptive empathy ~ Perspective Taking score

F(4,158) = 1.59, p = 0.18, R² = 0.04, Adj. R² = 0.01

| Name | Estimate | 5% | 95% | t(158) | p |
| --- | --- | --- | --- | --- | --- |
| (Intercept) | 60.20 | 47.23 | 73.17 | 7.68 | 0.00 |
| PT | 0.38 | -0.00 | 0.77 | 1.64 | 0.10 |
| age | 0.02 | -0.13 | 0.17 | 0.25 | 0.80 |
| gender - male | 3.15 | -1.05 | 7.35 | 1.24 | 0.22 |
| gender - non-binary | 24.46 | -1.46 | 50.39 | 1.56 | 0.12 |

Accuracy in social control ~ Perspective Taking score

F(4,158) = 2.69, p = 0.03, R² = 0.06, Adj. R² = 0.04

| Name | Estimate | 5% | 95% | t(158) | p |
| --- | --- | --- | --- | --- | --- |
| (Intercept) | 76.92 | 63.65 | 90.18 | 9.59 | 0.00 |
| PT | 0.26 | -0.13 | 0.66 | 1.10 | 0.27 |
| age | -0.26 | -0.41 | -0.11 | -2.81 | 0.01 |
| gender - male | 3.02 | -1.28 | 7.32 | 1.16 | 0.25 |
| gender - non-binary | 20.07 | -6.45 | 46.60 | 1.25 | 0.21 |

Accuracy in non-social control ~ Perspective Taking score

F(4,158) = 1.55, p = 0.19, R² = 0.04, Adj. R² = 0.01

| Name | Estimate | 5% | 95% | t(158) | p |
| --- | --- | --- | --- | --- | --- |
| (Intercept) | 85.77 | 70.69 | 100.86 | 9.41 | 0.00 |
| PT | -0.36 | -0.81 | 0.09 | -1.33 | 0.19 |
| age | -0.16 | -0.33 | 0.02 | -1.51 | 0.13 |
| gender - male | 4.34 | -0.54 | 9.23 | 1.47 | 0.14 |
| gender - non-binary | 8.49 | -21.67 | 38.64 | 0.47 | 0.64 |

Accuracy in Δ(adaptive empathy – non-social control) ~ Perspective Taking score

F(4,158) = 1.73, p = 0.15, R² = 0.04, Adj. R² = 0.02

| Name | Estimate | 5% | 95% | t(158) | p |
| --- | --- | --- | --- | --- | --- |
| (Intercept) | -25.57 | -45.09 | -6.06 | -2.17 | 0.03 |
| PT | 0.75 | 0.16 | 1.33 | 2.12 | 0.04 |
| age | 0.18 | -0.04 | 0.41 | 1.33 | 0.18 |
| gender - male | -1.19 | -7.51 | 5.13 | -0.31 | 0.76 |
| gender - non-binary | 15.98 | -23.03 | 54.98 | 0.68 | 0.50 |

Accuracy in adaptive empathy ~ Emotion Contagion score

F(4,158) = 1.18, p = 0.32, R² = 0.03, Adj. R² = 0.00

| Name | Estimate | 5% | 95% | t(158) | p |
| --- | --- | --- | --- | --- | --- |
| (Intercept) | 78.06 | 66.28 | 89.83 | 10.97 | 0.00 |
| EC | -0.52 | -1.36 | 0.31 | -1.04 | 0.30 |
| age | 0.03 | -0.12 | 0.18 | 0.30 | 0.77 |
| gender - male | 2.77 | -1.51 | 7.04 | 1.07 | 0.29 |
| gender - non-binary | 24.44 | -1.75 | 50.62 | 1.54 | 0.12 |

Accuracy in social control ~ Emotion Contagion score

F(4,158) = 2.38, p = 0.05, R² = 0.06, Adj. R² = 0.03

| Name | Estimate | 5% | 95% | t(158) | p |
| --- | --- | --- | --- | --- | --- |
| (Intercept) | 85.65 | 73.62 | 97.69 | 11.78 | 0.00 |
| EC | -0.07 | -0.92 | 0.78 | -0.14 | 0.89 |
| age | -0.25 | -0.41 | -0.10 | -2.74 | 0.01 |
| gender - male | 2.99 | -1.38 | 7.37 | 1.13 | 0.26 |
| gender - non-binary | 18.87 | -7.89 | 45.63 | 1.17 | 0.25 |

Accuracy in non-social control ~ Emotion Contagion score

F(4,158) = 2.86, p = 0.03, R² = 0.07, Adj. R² = 0.04

| Name | Estimate | 5% | 95% | t(158) | p |
| --- | --- | --- | --- | --- | --- |
| (Intercept) | 93.36 | 79.94 | 106.77 | 11.51 | 0.00 |
| EC | -1.50 | -2.45 | -0.55 | -2.62 | 0.01 |
| age | -0.18 | -0.36 | -0.01 | -1.79 | 0.07 |
| gender - male | 3.02 | -1.86 | 7.89 | 1.02 | 0.31 |
| gender - non-binary | 16.80 | -13.03 | 46.64 | 0.93 | 0.35 |

Accuracy in Δ(adaptive empathy – non-social control) ~ Emotion Contagion score

F(4,158) = 1.01, p = 0.40, R² = 0.03, Adj. R² = 0.00

| Name | Estimate | 5% | 95% | t(158) | p |
| --- | --- | --- | --- | --- | --- |
| (Intercept) | -15.30 | -33.08 | 2.49 | -1.42 | 0.16 |
| EC | 0.98 | -0.28 | 2.24 | 1.29 | 0.20 |
| age | 0.21 | -0.01 | 0.44 | 1.55 | 0.12 |
| gender - male | -0.25 | -6.72 | 6.21 | -0.06 | 0.95 |
| gender - non-binary | 7.63 | -31.91 | 47.18 | 0.32 | 0.75 |

Accuracy in adaptive empathy ~ Proximal Responsivity score

F(4,158) = 1.76, p = 0.14, R² = 0.04, Adj. R² = 0.02

| Name | Estimate | 5% | 95% | t(158) | p |
| --- | --- | --- | --- | --- | --- |
| (Intercept) | 60.27 | 48.39 | 72.15 | 8.39 | 0.00 |
| PRR | 0.95 | 0.09 | 1.81 | 1.82 | 0.07 |
| age | 0.02 | -0.13 | 0.17 | 0.20 | 0.84 |
| gender - male | 4.43 | 0.09 | 8.77 | 1.69 | 0.09 |
| gender - non-binary | 22.72 | -3.06 | 48.50 | 1.46 | 0.15 |

Accuracy in social control ~ Proximal Responsivity score

F(4,158) = 2.73, p = 0.03, R² = 0.06, Adj. R² = 0.04

| Name | Estimate | 5% | 95% | t(158) | p |
| --- | --- | --- | --- | --- | --- |
| (Intercept) | 92.19 | 80.01 | 104.36 | 12.53 | 0.00 |
| PRR | -0.62 | -1.51 | 0.26 | -1.16 | 0.25 |
| age | -0.24 | -0.40 | -0.09 | -2.63 | 0.01 |
| gender - male | 2.26 | -2.19 | 6.70 | 0.84 | 0.40 |
| gender - non-binary | 18.26 | -8.15 | 44.68 | 1.14 | 0.25 |

Accuracy in non-social control ~ Proximal Responsivity score

F(4,158) = 3.55, p = 0.01, R² = 0.08, Adj. R² = 0.06

| Name | Estimate | 5% | 95% | t(158) | p |
| --- | --- | --- | --- | --- | --- |
| (Intercept) | 96.92 | 83.40 | 110.44 | 11.86 | 0.00 |
| PRR | -1.83 | -2.82 | -0.85 | -3.09 | 0.00 |
| age | -0.14 | -0.31 | 0.03 | -1.36 | 0.18 |
| gender - male | 1.94 | -3.00 | 6.87 | 0.65 | 0.52 |
| gender - non-binary | 9.67 | -19.67 | 39.01 | 0.55 | 0.59 |

Accuracy in Δ(adaptive empathy – non-social control) ~ Proximal Responsivity score

F(4,158) = 3.94, p = 0.00, R² = 0.09, Adj. R² = 0.07

| Name | Estimate | 5% | 95% | t(158) | p |
| --- | --- | --- | --- | --- | --- |
| (Intercept) | -36.65 | -54.10 | -19.20 | -3.48 | 0.00 |
| PRR | 2.78 | 1.52 | 4.05 | 3.63 | 0.00 |
| age | 0.16 | -0.06 | 0.38 | 1.19 | 0.24 |
| gender - male | 2.50 | -3.87 | 8.86 | 0.65 | 0.52 |
| gender - non-binary | 13.05 | -24.81 | 50.91 | 0.57 | 0.57 |

Accuracy in adaptive empathy ~ Peripheral Responsivity score

F(4,158) = 1.62, p = 0.17, R² = 0.04, Adj. R² = 0.0

| Name | Estimate | 5% | 95% | t(158) | p |
| --- | --- | --- | --- | --- | --- |
| (Intercept) | 60.84 | 48.64 | 73.05 | 8.25 | 0.00 |
| PER | 0.88 | 0.01 | 1.74 | 1.67 | 0.10 |
| age | 0.04 | -0.10 | 0.19 | 0.49 | 0.62 |
| gender - male | 4.43 | 0.06 | 8.79 | 1.68 | 0.10 |
| gender - non-binary | 20.42 | -5.47 | 46.30 | 1.31 | 0.19 |

Accuracy in social control ~ Peripheral Responsivity score

F(4,158) = 2.43, p = 0.05, R² = 0.06, Adj. R² = 0.03

| Name | Estimate | 5% | 95% | t(158) | p |
| --- | --- | --- | --- | --- | --- |
| (Intercept) | 81.66 | 69.13 | 94.20 | 10.78 | 0.00 |
| PER | 0.25 | -0.64 | 1.14 | 0.47 | 0.64 |
| age | -0.25 | -0.40 | -0.10 | -2.69 | 0.01 |
| gender - male | 3.40 | -1.08 | 7.89 | 1.26 | 0.21 |
| gender - non-binary | 18.04 | -8.54 | 44.61 | 1.12 | 0.26 |

Accuracy in non-social control ~ Peripheral Responsivity score

F(4,158) = 2.34, p = 0.06, R² = 0.06, Adj. R² = 0.03

| Name | Estimate | 5% | 95% | t(158) | p |
| --- | --- | --- | --- | --- | --- |
| (Intercept) | 91.38 | 77.31 | 105.45 | 10.75 | 0.00 |
| PER | -1.33 | -2.33 | -0.33 | -2.20 | 0.03 |
| age | -0.19 | -0.36 | -0.01 | -1.78 | 0.08 |
| gender - male | 2.44 | -2.59 | 7.48 | 0.80 | 0.42 |
| gender - non-binary | 13.36 | -16.47 | 43.19 | 0.74 | 0.46 |

Accuracy in Δ(adaptive empathy – non-social control) ~ Peripheral Responsivity score

F(4,158) = 2.62, p = 0.04, R² = 0.06, Adj. R² = 0.04

| Name | Estimate | 5% | 95% | t(158) | p |
| --- | --- | --- | --- | --- | --- |
| (Intercept) | -30.53 | -48.71 | -12.35 | -2.78 | 0.01 |
| PER | 2.21 | 0.91 | 3.50 | 2.82 | 0.01 |
| age | 0.23 | 0.01 | 0.45 | 1.71 | 0.09 |
| gender - male | 1.98 | -4.52 | 8.49 | 0.50 | 0.61 |
| gender - non-binary | 7.05 | -31.49 | 45.60 | 0.30 | 0.76 |

## Descriptive statistics of QCAE questionnaire scales and subscales

|  | AE | CE | EC | OS | PER | PRR | PT | TE |
| --- | --- | --- | --- | --- | --- | --- | --- | --- |
| mean (sd) | 34.9 (5.6) | 58.7 (8.6) | 11.5 (2.5) | 27.8 (4.4) | 11.3 (2.4) | 12.0 (2.4) | 30.9 (5.3) | 93.6 (11.9) |

## Descriptive statistics of task duration per block (in minutes)

| block | mean (sd) | min | max |
| --- | --- | --- | --- |
| adaptive empathy | 4 (1.68) | 1.65 | 13.7 |
| non-social control | 1( 0.49 ) | 0.7 | 6.23 |
| social control | 3 (1.99 ) | 1.47 | 19.4 |

## Correlation with learning performance in adaptive empathy condition

We tested whether emotion regulation strategies personal preferences were correlated with accuracy in adaptive empathy condition, based on the preference of the target: cognitive reappraisal or emotional suppression. Two separate linear regression analyses were conducted entering ERQ scores as the potential predictor variable and learning accuracy at the consequent condition as the single dependent variable. The analyses revealed that personal emotional regulation preferences were positively (CR) and negatively (ES) associated with performance in the corresponding blocks, however, insignificantly, CR: (β = 2.3, R² = 0.04, t(84) = 1.8, p = 0.08); ES: (β = -0.58, R² = 0, t(75) = -0.37, p = 0.71). (see figure Supplementary Figure 1 and Supplementary Figure 2)


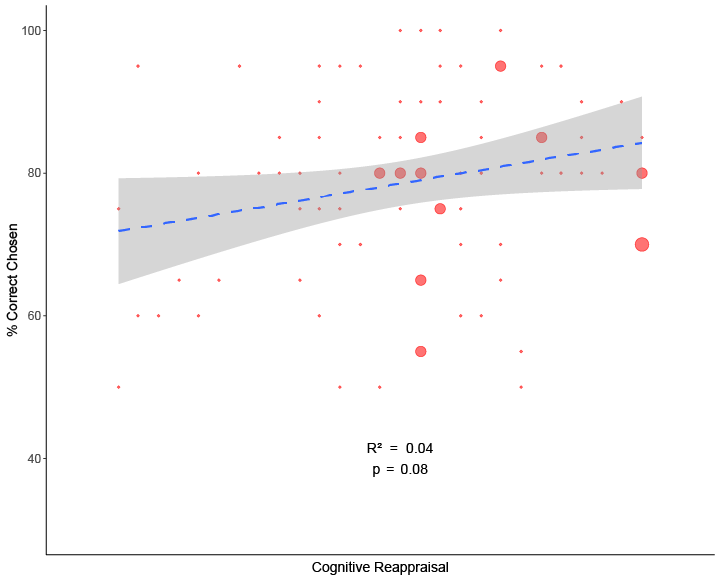


Supplementary Figure 1 – Correlation of Cognitive Reappraisal tendency with learning accuracy

When the preferred strategy of the target was cognitive reappraisal, the learning accuracy in adaptive empathy condition was marginally predicted by personal preferences of cognitive reappraisal.


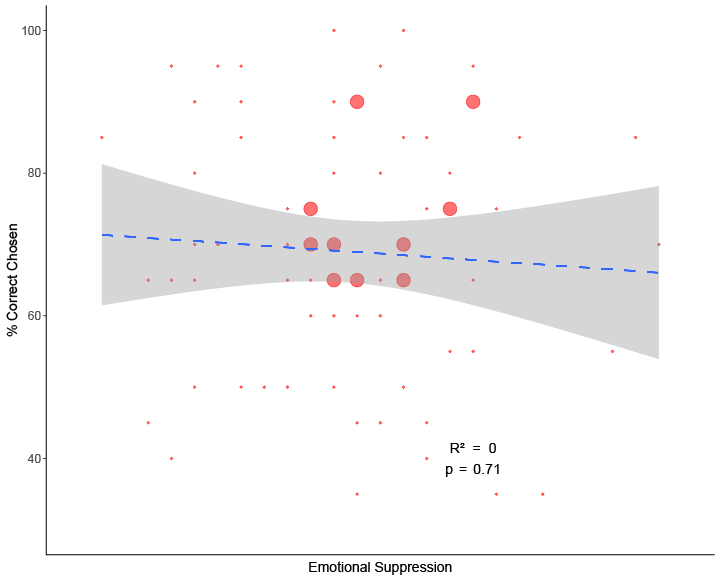


Supplementary Figure 2 – Correlation of Emotional Suppression tendency with learning accuracy

When the preferred strategy of the target was emotional suppression, the learning accuracy in adaptive empathy condition was not predicted by personal preferences of emotional suppression

## Additional analysis and graphical tools

Additional R packages for figures production used under R version 4.0.1. were: tidyverse (3), lubridate (4), cowplot (5), gtsummary (6), kableExtra (7), wesanderson (8), svglite (9), ggstatsplot (10), ggpubr (11).

# References

1. Gross JJ, John OP. Individual differences in two emotion regulation processes: Implications for affect, relationships, and well-being. *J Pers Soc Psychol* (2003) **85**:348–362. doi:10.1037/0022-3514.85.2.348

2. Reniers RLEP, Corcoran R, Drake R, Shryane NM, Völlm BA. The QCAE: A questionnaire of cognitive and affective empathy. *J Pers Assess* (2011) **93**:84–95. doi:10.1080/00223891.2010.528484

3. Wickham H, Averick M, Bryan J, Chang W, McGowan LD, François R, Grolemund G, Hayes A, Henry L, Hester J, et al. Welcome to the {tidyverse}. *J Open Source Softw* (2019) **4**:1686. doi:10.21105/joss.01686

4. Grolemund G, Wickham H. Dates and Times Made Easy with {lubridate}. *J Stat Softw* (2011) **40**:1–25. Available at: https://www.jstatsoft.org/v40/i03/

5. Wilke CO. cowplot: Streamlined Plot Theme and Plot Annotations for “ggplot2.” (2020) Available at: https://cran.r-project.org/package=cowplot

6. Sjoberg DD, Curry M, Hannum M, Whiting K, Zabor EC. gtsummary: Presentation-Ready Data Summary and Analytic Result Tables. (2021) Available at: https://cran.r-project.org/package=gtsummary

7. Zhu H. kableExtra: Construct Complex Table with “kable” and Pipe Syntax. (2020) Available at: https://cran.r-project.org/package=kableExtra

8. Ram K, Wickham H. wesanderson: A Wes Anderson Palette Generator. (2018) Available at: https://cran.r-project.org/package=wesanderson

9. Wickham H, Henry L, Pedersen TL, Luciani TJ, Decorde M, Lise V. svglite: An “SVG” Graphics Device. (2020) Available at: https://cran.r-project.org/package=svglite

10. Patil I. {ggstatsplot}: “ggplot2” Based Plots with Statistical Details. *CRAN* (2018) doi:10.5281/zenodo.2074621

11. Kassambara A. ggpubr: “ggplot2” Based Publication Ready Plots. (2020) Available at: https://cran.r-project.org/package=ggpubr
